# Does Short-Term Dietary Omega-3 Fatty Acid Supplementation Influence Brain Hippocampus Gene Expression of Zinc Transporter-3?

**DOI:** 10.3390/ijms160715800

**Published:** 2015-07-13

**Authors:** Nur Farhana Ahmad Sopian, Mokrish Ajat, Nurul’ Izzati Shafie, Mohd Hezmee Mohd Noor, Mehdi Ebrahimi, Mohamed Ali Rajion, Goh Yong Meng, Hafandi Ahmad

**Affiliations:** Department of Veterinary Preclinical Sciences, Faculty of Veterinary Medicine, Universiti Putra Malaysia, 43400 UPM Serdang, Selangor, Malaysia; E-Mails: nurfarhana4361@gmail.com (N.F.A.S.); mokrish@upm.edu.my (M.A.); nurul_izzati90@ymail.com (N.I.S.); hezmee@upm.edu.my (M.H.M.N.); mehdiebrahimii@gmail.com (M.E.); mohdali@upm.edu.my (M.A.R.); ymgoh@upm.edu.my (G.Y.M.)

**Keywords:** omega-3 fatty acid, brain gene expression, *ZnT3*, cognitive function

## Abstract

Dietary omega-3 fatty acids have been recognized to improve brain cognitive function. Deficiency leads to dysfunctional zinc metabolism associated with learning and memory impairment. The objective of this study is to explore the effect of short-term dietary omega-3 fatty acids on hippocampus gene expression at the molecular level in relation to spatial recognition memory in mice. A total of 24 male BALB/c mice were randomly divided into four groups and fed a standard pellet as a control group (CTL, *n* = 6), standard pellet added with 10% (*w*/*w*) fish oil (FO, *n* = 6), 10% (*w*/*w*) soybean oil (SO, *n* = 6) and 10% (*w*/*w*) butter (BT, *n* = 6). After 3 weeks on the treatment diets, spatial-recognition memory was tested on a Y-maze. The hippocampus gene expression was determined using a real-time PCR. The results showed that 3 weeks of dietary omega-3 fatty acid supplementation improved cognitive performance along with the up-regulation of α-synuclein, calmodulin and transthyretin genes expression. In addition, dietary omega-3 fatty acid deficiency increased the level of *ZnT3* gene and subsequently reduced cognitive performance in mice. These results indicate that the increased the *ZnT3* levels caused by the deficiency of omega-3 fatty acids produced an abnormal zinc metabolism that in turn impaired the brain cognitive performance in mice.

## 1. Introduction

Dietary omega-3 fatty acids have important roles in the regulation of brain gene expression associated with brain cognitive function [1,2]. As has been reported previously, numerous genes were detected as overexpressed in rodents fed omega-3 fatty acids [3,4]. The findings show that the expression of brain genes can be regulated by polyunsaturated fatty acid (PUFA) supplementation, using cDNA microarray analysis and real-time quantitative RT-QPCR. For example, transthyretin gene participating in signal transduction processes were overexpressed in rat brains receiving a docosahexaenoic acid (DHA) enriched diet for one month [5]. Similarly, transthyretin gene expression was increased in the hippocampus of rats when they were given fish oil for 4 weeks [2]. In other study, gene coding for α- and γ-synuclein also were overexpressed in response to the diets rich in omega-3 fatty acids for 9 weeks [6]. The transcript levels of calmodulin also up-regulated to the same extent by the different dietary supplementation with α-linolenic and linoleic acid for 4 weeks [7]. The DHA enriched diet for 2 weeks in rats increased hippocampal expression of molecules involved in brain-derived neurotrophic factor (BDNF) signaling such as calmodulin kinase II and activated Akt [8]. Generally, these findings suggest that the length of feeding time on omega-3 fatty acids might be a factor affecting the gene expression profile.

Zinc is an essential nutrient and has important roles in controlling the function of genetic systems especially neurotransmission [9] and signal transduction in the brain hippocampus [10]. Although zinc is critically important in a number of physiological processes, in excess amounts it is potently neurotoxic causing generalized cell death or zinc homeostasis [11]. Indeed, to prevent these unnecessary functions, suitable cellular concentrations of zinc must be maintained. This involves the transport of the zinc into and out of the cell, as well as into various organelles by zinc transporters [12]. The *ZnT3*, a member of the zinc transporter family is a putative transporter of zinc into synaptic vesicles of neurons and found abundantly in brain areas such as hippocampus and cortex [13]. Previous studies reported that over-expression of *ZnT3* caused abnormal zinc metabolism in the brain, which may contribute to neuronal cell injury and apoptosis [14]. Therefore, cellular zinc concentrations need to be tightly controlled as an altered zinc metabolism or dyshomeostatis can be a contributing factor in neurodegenerative processes associated with memory loss and formation of amyloid plaques as in Alzheimer disease (AD).

For the past three decades, omega-3 fatty acids have been recognized as essential nutrients for energy sources and structural components of the central nervous system. Their role in brain cognitive function has been well documented. For instance, we recently showed that cognitive impairment caused by an omega-3 fatty acid-deficient diet in the third generation mice can be prevented by dietary repletion with omega-3 fatty acids [15]. In fact, the sufficiency of this diet influenced many other cellular events such as gene regulation and transcription. However, inconsistent findings were observed an analytical interaction between omega-3 fatty acid and zinc metabolism associated with brain cognitive function. Therefore, the aim of the current study was to determine whether short-term dietary omega-3 fatty acid supplementation influences molecular interaction between brain gene expressions of zinc transporter-3 and cognitive function in mice.

## 2. Results

### 2.1. Body Weight, Food and Water Intake

Differences in mean body weights, food and water intake between the four dietary groups were not significant.

### 2.2. Y-Maze Performance

One-way ANOVA indicated a significant (*F*_3,20_ = 2.552, *p* < 0.05) interaction between treatment groups on the number of novel arm entries. The total number of novel arm entries was significantly (*p* < 0.05) higher in fish oil (FO) mice (6.78 ± 1.36 entries) than in butter (BT) mice (3.44 ± 0.42 entries) and control group (CTL) mice (3.96 ± 0.23 entries). However, the number of novel arm entries was similar between of soybean oil (SO) (4.78 ± 0.38 entries) and CTL (3.96 ± 0.23 entries) mice.

Furthermore, one-way ANOVA indicated a significant (F_3,20_ = 2.746, *p* < 0.05) interaction between treatment groups on time spent in the novel arm. The FO mice spent more time (*p* < 0.05) in the novel arm compared with the BT and CTL mice (75.94 ± 9.85 s compared with 42.01 ± 5.34 s and 52.90 ± 5.21 s). However, the time spent in the novel arm was similar for the SO (61.43 ± 8.21 s) and CTL (52.90 ± 5.21 s) mice.

### 2.3. Brain Hippocampus mRNA Expression

**Figure 1:**
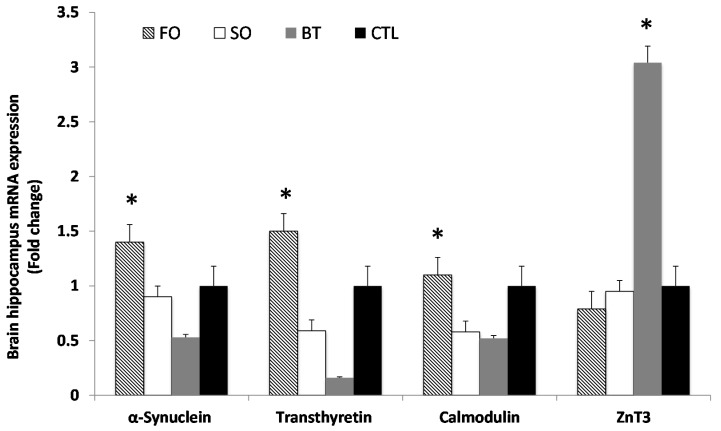
Expression of α-synuclein, transthyretin, calmodulin and *ZnT3* in the brain hippocampus of treatment groups compared to the CTL group. Results are normalized to the expression of GAPDH and β-actin. Treated samples were expressed relative to gene expression of the CTL group. Statistical analysis was performed using the Student’s *t*-test. Values indicated by * show significant differences compared with the CTL group at *p* < 0.05. FO, fish oil; SO, soybean oil; BT, butter; CTL, control.

As shown in Figure 1, expression of mRNA for α-synuclein (t_12df_ = −2.234, *p* < 0.05), calmodulin (t_12df_ = −2.234, *p* < 0.05) and transthyretin (t_12df_ = −2.234, *p* < 0.05) increased in the FO group compared with the SO, BT and CTL groups. However, the expression of mRNA for *ZnT3* (t_12df_ = −2.234, *p* < 0.05) was increased in the BT group compared with the other groups.

### 2.4. Brain Fatty Acids

As shown in Table 1, the total of omega-3 fatty acids in brain hippocampus was greater in the FO group (17.51 ± 1.07, *p* < 0.05) than SO, BT and CTL groups (15.64 ± 1.03, 14.74 ± 0.98 and 12.44 ± 0.89). The proportion of docosahexaenoic acid in the brain hippocampus was significantly higher in the FO group (14.47 ± 0.97, *p* < 0.05) than the other groups.

**Table 1:** Fatty acid profile (% of total identified fatty acids) of the brain hippocampus.

Treatments	FO	SO	BT	CTRL	SEM
C16:0 (Palmitic acid)	32.43	32.48	34.27	32.89	0.69
C16:1n-7 (Palmitoleic acid)	1.01	0.77	1.21	1.11	0.15
C18:0 (Stearic acid)	7.36	7.96	7.63	7.66	0.29
C18:1n-9 (Oleic acid)	29.09 ^b^	29.84 ^b^	29.86 ^b^	33.46 ^a^	0.75
C18:2n-6 (Linoleic acid)	5.72	5.55	5.82	6.44	0.16
C18:3n-6 (Linolenic acid)	0.83	0.99	0.77	0.75	0.06
C18:3n-3 (α-linolenic acid)	0.76	1.00	1.18	0.42	0.23
C20:1n-9 (Arachidic acid)	1.99	2.03	1.29	0.87	0.19
C20:4n-6 (Arachidonic acid)	1.03	1.03	1.29	1.36	0.12
C20:5n-3 (Eicoapentaenoic acid)	1.03	1.05	1.33	0.45	0.21
C22:4n-6 (Docosatetraenoic acid)	3.02	3.72	3.12	3.03	0.15
C22:5n-3 (Docosapentaenoic acid)	1.25	1.04	1.78	1.61	0.27
C22:6n-3 (Docosahexaenoic acid)	14.47 ^a^	12.55 ^ab^	10.44 ^b^	9.95 ^b^	0.73
TOTAL SFA	39.79	40.43	41.90	40.55	0.67
TOTAL MUFA	35.11	36.37	35.49	38.47	0.59
TOTAL n-6 PUFA	10.60	11.28	10.99	11.57	0.23
TOTAL n-3 PUFA	17.51 ^a^	15.64 ^ab^	14.74 ^ab^	12.44 ^b^	1.07
n-6:n-3 Ratio	0.63 ^b^	0.72 ^b^	0.84 ^ab^	0.93 ^a^	0.06

^a,b^ Different alphabets in the same row denote significant difference at *p* < 0.005. FO, fish oil; SO, soybean oil; BT, butter; CTL, control; SEM, standard error of the mean; SFA, saturated fatty acid; MUFA, monounsaturated fatty acid; PUFA, polyunsaturated fatty acid.

## 3. Discussion

The current study shows that short-term dietary omega-3 fatty acid supplementation improved spatial recognition memory in the Y-maze test and increased the hippocampal α-synuclein, calmodulin and transthyretin genes expression in the FO mice. These results are consistent with previous studies that have used different measures to assess visuospatial memory and gene profiles on the short-term effects [2,16,17]. The regulation of gene expression by dietary omega-3 fatty acids can be explained by the interactions fatty acids with the ligands (specific or non-specific) that bind to response factors acting on cis-regulatory elements of the gene, which finally turn on or off mRNA synthesis [3]. For instance, polyunsaturated fatty acids can directly interact with transcription factors, like peroxisome proliferator-activated receptors (PPAR) that directly modulate the expression of target genes [18]. In this study, the target gene such as α-synuclein was increased in the brain hippocampal of the FO mice compared to the SO and BT groups. In our study, we used soybean oil, which mammals commonly consume in their diets. The soybean oil contains 18:3n-3, which can convert into EPA (20:5n-3) and small amounts of DHA (22:6n-3) in the body [19]. In fact, the soybean oil contains less DHA but α-linolenic acid, which is the precursor of DHA and can also influence the brain fatty acid profile [20]. Thus, this finding suggests that that the fish oil rich in omega-3 fatty acid involved in the brain function mechanisms such as synaptic plasticity and signal transduction. Previous studies have reported that α-synuclein gene is specifically enriched at the synaptic contacts and play an important role in the development and maturation of some neurons or synaptogenesis [21,22]. Interestingly, this gene increased in the brains of songbirds and vocalization [23].

The calmodulin gene was also overexpressed in the hippocampus of mice on an omega-3 fatty acid supplemented diet. This result is consistent with the Y-maze performance of the FO mice, which showed higher total arm entries and time spent in novel arm. The increased level of calmodulin could have enhanced the signal transduction and communication between neurons during memory formation [24]. Additionally, the calmodulin gene is highly enriched in postsynaptic of the hippocampus and neocortex which are involved in the regulation of long-term potentiation (LTP) during learning processes [25,26]. Clearly, the participation of this gene in the presence of omega-3 fatty acids in the mice hippocampus resulted in the improved performance in the Y-maze task.

The decrease in the spatial-recognition memory of the BT mice is due to lower omega-3 fatty acid in the diet in parallel with decreased transthyretin gene expression. This result perhaps can be clarified in several contexts. Studies in transthyretin null mice revealed that absence of transthyretin reduced signs of depressive-like behavior [27] and delayed nerve regeneration in nerve injury conditions [28]. The absence of transthyretin is also related to memory dysfunction, an observation that is in accordance with the decline in cognitive performance that commonly occurs in AD [29]. Furthermore, transthyretin inducers such as *Ginkgo* extract or fish oil could be effective for the prevention of AD by deposition of amyloid beta peptides [2]. Thus, our results suggest that transthyretin has neuroprotective properties in cellular processes and their absence is related to deficits in learning and memory.

**Figure 2:**
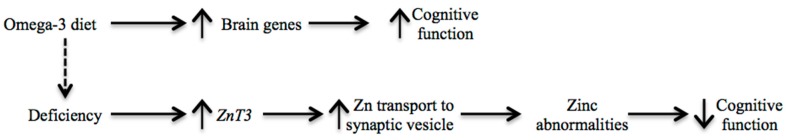
The potential molecular mechanism by which omega-3 fatty acid may interact with the zinc transporter in brain cognitive function.

In contrast, the *ZnT3* gene expression was up-regulated along with a reduced cognitive performance in the BT mice. These results are consistent with previous studies that showed that the *ZnT3* gene was up-regulated in hippocampus under dietary omega-3 restriction [30]. A possible explanation is that the over-expressed *ZnT3* gene caused abnormal zinc metabolism in the brain, which may involve the transport of zinc into synaptic vesicles during synaptic processes and neurotransmission. The postulated inter-relationship between omega-3 fatty acids is as depicted in Figure 2. Indeed, the DHA from PUFA induced a decrease in neuronal cell death through reduced *ZnT3* expression and zinc uptake [14]. Previous studies reported that an abnormal zinc metabolism in the brain contributed to cognitive impairment and beta-amyloid formation in the AD mice [31]. In fact, a high level of synaptic zinc will lead be toxicity in the brain neurons and consequently lead to neuronal injuries [32] and apoptotic cell death [33,34]. Thus, the up-regulation of *ZnT3* due to dietary omega-3 fatty acid deficiency caused cognitive impairment in the BT mice.

## 4. Experimental Section

### 4.1. Animals and Diet

Three-week old male BABL/c mice (*n* = 24) were purchased from the Animal Resource Centre, Faculty of Veterinary Medicine, Universiti Putra Malaysia, MALAYSIA. The mice were randomly divided into four groups and fed semi-synthetic diets that contained identical amounts of protein, carbohydrate, fat, vitamins, minerals and supplemented with either 10% (*w*/*w*) fish oil (FO, *n* = 6), 10% (*w*/*w*) soybean oil (SO, *n* = 6) and 10% (*w*/*w*) butter (BT, *n* = 6). The standard pellet represented the control diet (CTL, *n* = 6). After 3 weeks on the treatment diets, all animals were tested for cognitive function by the Y-maze test. The brain gene expression and brain fatty acid profile were determined at the end of experiment. All testing related to animal care and handling was approved by the Institutional Animal Care and Use Committee (IACUC) of the Faculty of Veterinary Medicine, Universiti Putra Malaysia, Malaysia (UPM/IACUC/2014/084, 10 January 2014).

### 4.2. Y-Maze Test

The cognitive function of mice was tested by using the Y-maze test, as described previously [15]. The Y-maze had three identical arms of equal size: the start arm, in which the mouse is first placed (always open); the familiar arm (always open); and the novel arm, which was blocked during the first trial but open during the second trail. Different visual cues were placed on the wall at the end of each arm of the maze. The Y-maze testing consisted of two trials separated by an interval of 1 h. The first trial was 10 min in duration and allowed the mouse to explore only two arms (the start and familiar arms) of the maze, with the third arm (novel arm) blocked. After 1 h, the second trial was conducted; mice were placed in the same starting arms in trial 1, with free access to all three arms for 5 min. Trials were recorded using a ceiling-mounted camera. Recordings were then monitored to count the number of entries and the time spent in each arm. Y-maze performance was favorable when the number of entries and time spent in the novel arm were greater than those in the other arms. The total number of arm entries and time (in seconds) spent in the novel arm are indicator of spatial working memory [35].

### 4.3. RNA Extraction and Real-Time RT-PCR

At the end of 4 weeks, animals were euthanized and tissue samples of the hippocampus (~20 mg) were collected and extracted using a RNeasy Mini Kit from Qiagen (QIAGEN GmbH, Hilden, Germany). The qRT-PCR assays for relative quantification of the respective genes α-synuclein, transthyretin, calmodulin, and *ZnT3* expression in each sample were performed using a commercial SYBR Green One-Step RT-PCR master mix System (Applied Biosystems, Petaling Jaya, Selangor, Malaysia). The PCR primers set for the all genes were adapted from a previous study and were synthesized by First Base (Selangor, Malaysia) according to the cDNA sequences obtained from the Gene Bank™ database for mice. The relative expression ratios were normalized to β-actin and GADPH. The sequences of primers were as follows: β-actin (NM_007393), forward primer, 5′-TTCAACACCCCAGCCATGT-3′, reverse primer 5′-GCATACAGGGACAACACAGCC-3′; GADPH (NM_008084), forward primer, 5′-ACCCAGAAGACTGTGGATGG-3′, reverse primer 5′-TTCAGCTCTGGGATGACCTT-3′; α-synuclein (NM_001042451), forward primer, 5′-GGCGACGTGAAGGAGCCAGGG-3′, reverse primer, 5′-CAGCGAAAGGAAAGCCGAGTGATGTACT-3′; transthyretin (NM_013697), forward primer, 5′-GGCTCACCACAGATGAGAAGTTC-3′, reverse primer, 5′-ACAAATGGGAGCTACTGCTTTGGC-3′; calmodulin (NM_054805), forward primer, 5′-GCACCATCACAACCAAGGA-3′, reverse primer, 5′-CCATTCCCATCCTTGTCAAA-3′ and *ZnT3* (NM_011773), forward primer, 5′-GGAGGTGGTTGGTGGGTATTTAGC-3′, reverse primer, 5′-GATGGAGATCATGGGTTGCTCG-3′. During initial optimization, the exact primer concentrations and PCR conditions were determined. Following optimization experiments, the assays of a total volume of 20 µL reaction mix consisting of an equal concentration of RNA, 10 µL of EXPRESS SYBR^®^ GreenER™ qPCR SuperMix Universal (Bio-diagnostics, Petaling Jaya, Selangor, Malaysia) with 10 nM of each forward and reverse primer were performed. The reactions were set up in MicroAmp^®^ fast 8-tube strip covered with MicroAmp^®^ optical 8-cap strips and were run on an ABI Step-One-Plus™ Real-Time PCR Systems (Applied Biosystems). The cycling conditions were at 45 °C for 45 min, followed by 40 cycles of 95 °C for 5 min, 45 cycles of 30 s at 95 °C, 1 min at 59 °C, and 30 s at 72 °C. To check the specificity of the amplified products, a melting curve analysis was performed immediately following the completion of the PCR. All samples were amplified on the same plate for every primer pair to ensure equal amplification conditions, and no-template controls with water instead of RNA templates were also included as negative controls. Each sample was then run in triplicate and their results were documented as cycle threshold (*C*_t_) values. For each sample, *C*_t_ values of the genes were averaged and its relative expression levels to the housekeeping genes were calculated using the comparative ∆*C*_t_ method. The housekeeping genes used were shown to represent the best reference gene for brain tissues.

### 4.4. Brain Fatty Acid Analysis

A brain tissue sample of the hippocampus (approximately 0.5 g) was mixed with 40 mL of chloroform-methanol (2:1, *v*/*v*) containing butylated hydroxytoluene as antioxidant according to [36] modified by [37]. Then, fatty acids methyl esters (FAME) were prepared using 0.66 N potassium hydroxide (KOH) in methanol and 14% methanolic boron trifluoride (BF_3_) (Sigma Chemical Co., St. Louis, MO, USA). The FAME were separated with an Agilent 6890A Series GC system (Agilent Technologies, Palo Alto, CA, USA) using a 30 m × 0.25 mm ID (0.20 µm film thickness) Supelco SP-2330 capillary column (Supelco, Inc.; Bellefonte, PA, USA). The fatty acid proportions are expressed as percentage of total identified fatty acids. One microlitre of FAME was injected by an auto sampler into the chromatograph, equipped with a split injector and a flame ionization detector (FID) detector. The injector temperature was programmed at 250 °C and the detector temperature was 300 °C. The column temperature program initiated runs at 100 °C, for 2 min, warmed to 170 °C at 10 °C/min, held for 2 min, warmed to 200 °C at 7.5 °C/min, and then held for 20 min to facilitate optimal separation. A reference standard (mix C4–C24 methyl esters; Sigma-Aldrich, Inc.; St. Louis, MO, USA) containing FAME, methyl palmitate, methyl stearate, methyl oleate, methyl linoleate, methyl linolenate, gamma linolenate, methyl arachidonate, methyl eicosapentaenoate, and methyl docosahexaenoate were used to determine recoveries and correction factors for the determination of individual fatty acid composition. The fatty acid concentrations are expressed as percentage of total identified fatty acids.

### 4.5. Statistical Analysis

One-way ANOVA with repeated measures on one variable followed by a post hoc least significant difference test (Statistica 7, StatSoft, Tulsa, OK, USA) was used to assess performance in the Y-maze test between the groups. The expression of mRNA were analysed by an unpaired Student’s *t*-test. All data are reported as mean ± SEM; statistical significance was defined as a *p* value of less than 0.05.

## 5. Conclusions

Overall, our current results suggest that short-term dietary omega-3 fatty acid supplementation increased certain brain gene expression and consequently improved brain cognitive function in mice. It was proven here that a short-term dietary omega-3 fatty acid deficiency increased the levels of *ZnT3* gene expression, which affected the cognitive performance in mice. It is recommended to investigate further if the up-regulation of *ZnT3* or other zinc transporters would result in efflux and influx of cellular zinc causing generalized neuronal cell damage. Thus, the involvement of several genes induced by dietary omega-3 fatty acid through molecular alterations of the membrane phospholipids are relevant to the synaptic mechanisms such as synaptogenesis and signal transduction processes that mediate the formation of learning and memory.
